# Editorial: Pathologic Conditions of the Human Nervous and Muscular Systems Associated with Mutant Chaperones: Molecular and Mechanistic Aspects

**DOI:** 10.3389/fmolb.2018.00014

**Published:** 2018-02-16

**Authors:** Alberto J. L. Macario, Everly Conway de Macario

**Affiliations:** ^1^Department of Microbiology and Immunology, School of Medicine, University of Maryland at Baltimore-Institute of Marine and Environmental Technology (IMET), Columbus Center, Baltimore, MD, United States; ^2^Euro-Mediterranean Institute of Science and Technology (IEMEST), Palermo, Italy

**Keywords:** Genetic Chaperonopathies, Hsp60/CCT/BBS, Hsp40/DnaJ, small-Hsp, Indirect/Secondary Chaperonopathies, Chaperonotherapy

This Research Topic focuses on genetic chaperonopathies (http://www.chaperones-pathology.org) affecting predominantly muscles and nerves. It should be useful to professionals in the medical and closely related sciences, particularly physicians and clinical pathologists in practice and in applied and basic research. These were the objectives outlined for the Research Topic (see About this Research Topic). By examining the current series of articles with their figures and tables, the references cited, and the diversity of contributors, one may say that the objectives have been attained at least in as much as the field covered is vast and the materials discussed varied. This cornucopia of information we hope will reach many for the ultimate benefit of a large number of patients and their families.

One of the important benefits of the notion that a chaperone can cause disease is that it alerts the researcher and the physician to the possibility that there is a pathogenic pathway, different from the others known to occur in any particular patient, which may be amenable to specific diagnostic and treatment procedures. This notion, in turn, engenders the idea that chaperones can be therapeutic targets or agents. For example, patients with chaperonopathies by defect may benefit from positive chaperonotherapy, namely from procedures aiming at correcting the deficiency by gene replacement or chaperone administration. Since developments in chaperonotherapy must be rooted in basic and clinical research, information in this Research Topic should be instrumental for investigators to find ways of preventing and curing chaperonopathies. Contributions are here presented in the order of their publication. All Figures and Tables cited in this Editorial can be found in the articles being discussed.

Accumulation of SOD1 (Superoxide Dismutase 1) mutant protein in neurons is pathogenic in some forms of amyotrophic lateral sclerosis (*SOD1*-ALS). Anzai et al. describe their attempts to find drugs that specifically interact with the misfolded SOD1 mutant molecules and suppress their pathologic aggregation. Their findings provide clues on which molecular structures should be further tested and modified to obtain efficacious drugs with minimal side effects.

Missense mutations in the *HSP60* gene cause SPG13 and MitCHAP-60 chaperonopathies, as discussed by Bross and Fernandez-Guerra. Figure 1 and Table 1 are excellent teaching tools that can be used to spread knowledge not only on genetic HSP60 chaperonopathies, but also to explain what might happen to other chaperones if mutated at critical sites.

The article by Sorge and Brancaccio provides a useful review on the roles played by chaperones in the stressed heart, focusing on Melusin, and opening eyes as to possible developments in the treatment of heart diseases centered on this chaperone. Melusin is a muscle specific chaperone protein that induces adaptive hypertrophy and cardiomyocyte survival under stress.

Ruggieri et al. discuss DNAJB6 chaperonopathies and present an overview of the various mutations and phenotypes identified thus far (Table 1), focusing on limb girdle muscular dystrophy 1D. Fascinating questions that should stimulate future research emerge from the information provided, pertaining to tissue pathology differential distribution, mutations in DNAJ6 domains other than G/F, and chaperonotherapy using DNAJ gene/protein.

The contribution by Bie et al. represents the first ever description of an HSP10 chaperonopathy and offers a variety of starting points for future research not only on HSP10, but also on its functional partner, HSP60, and on mitochondrial disorders.

Tang and Xia review the pathogenic mutations of the human protein p97, including Multisystem Proteinopathies, Familial Amyotrophic Lateral Sclerosis, and Charcot-Marie-Tooth Disease Type 2Y (Table 1). Because of its many functions in various cellular locales, p97 interacts with a variety of co-factors and adaptors in a complex matrix, which is very difficult to elucidate. This article is very stimulating because it explains clearly the facts and the problems and suggests possible ways to advance the field.

Lupo et al. deal with pathogenic mutations in four chaperone genes (*DNAJB, HSPB1, HSPB3*, and *HSPB8*) that cause distal hereditary motor neuropathies (dHMN). Table 1 is a useful compendium of mutations, phenotypes, and bibliography. The connection between chaperones and chaperonopathies and the distinctive features of chaperonopathies as compared with proteinopathies are clearly explained.

Experimental models are necessary to study the molecular mechanisms that in chaperonopathies produce the abnormalities observed in tissues and organs, since work with human specimens is very limited. Conway de Macario et al. report on experimental models using archaea that possess chaperones very similar to those of humans. Studies on CCT chaperonopathies are reported along with a list of genes that may be affected and pathogenic mutations already identified (Tables 1, 2).

Pelizaeus-Merzbacher disease (PMD) is a hypomyelinating leukodystrophy associated with mutations in *PLP1*, which encodes a major myelin protein. Mutations in genes encoding membrane proteins (nicely explained in Figure 1, in the article by Inoue) may lead to the production of abnormal polypeptides that accumulate in the ER of the myelin-producing oligodendrocytes and, thereby, elicit the unfolded protein response (UPR), involving ER chaperones (explained in Figure 2). This would be an example of a secondary chaperonopathy, namely the mutated gene does not encode a chaperone but, ultimately, has an impact on chaperones, which become pathogenic. Suggestions for development of chaperonotherapy for PMD and other leukodystrophies are offered.

The article by Lanfranco et al. discusses Spinal Muscular Atrophy (SMA) and some of the molecular mechanisms of it that point toward a failure in chaperoning activity. The disease is directly associated with decreased levels of the Survival Motor Neuron (SMN) protein. This protein is part of a complex that functions as a molecular chaperone, interacting with and assisting in the assembly of small ribonuleoproteins (snRNPs). It is concluded that SMA may be considered a chaperonopathy and, consequently, amenable to chaperonotherapy. This is important because other disorders, such as adult-onset amyotrophic lateral sclerosis (ALS), show disturbance of snRNP assembly.

Bardet-Biedl syndrome (BBS) comprises a group of genetic diseases called ciliopathies since cell cilia are defective. The article by Alvarez-Satta et al. presents a subset of BBS as chaperonopathies because of the 21 or so genes whose mutations are currently known to be associated with BBS, three (BBS6, 10, and 12) belong to the family of CCT chaperonins. Other facts that argue in favor of considering some BBS as chaperonopathies are that over 50% of all BBS-affected families carry a pathogenic mutation in one of these three genes, and patients with mutations in any one of these three genes are more severely ill.

## Author contributions

All authors listed have made a substantial, direct and intellectual contribution to the work, and approved it for publication.

### Conflict of interest statement

The authors declare that the research was conducted in the absence of any commercial or financial relationships that could be construed as a potential conflict of interest.

